# Gestational Diabetes Mellitus: Association with Maternal and Neonatal Complications

**DOI:** 10.3390/medicina59122096

**Published:** 2023-11-29

**Authors:** Rebecca Karkia, Tara Giacchino, Saadia Shah, Andrew Gough, Ghada Ramadan, Ranjit Akolekar

**Affiliations:** 1 Medway Fetal and Maternal Medicine Centre, Medway NHS Foundation Trust, Gillingham ME7 5NY, UK; 2 Women’s Care Group, Department of Obstetrics, Medway NHS Foundation Trust, Gillingham ME7 5NY, UK; 3 Department of Diabetes and Endocrinology, Medway NHS Foundation Trust, Gillingham ME7 5NY, UK; 4 Oliver Fisher Neonatal Unit, Medway NHS Foundation Trust, Gillingham ME7 5NY, UK; 5 Institute of Medical Sciences, Canterbury Christ Church University, Chatham, Kent ME4 4UF, UK

**Keywords:** gestational diabetes mellitus, diabetes mellitus, adverse pregnancy outcome

## Abstract

*Background and objectives*: Gestational diabetes mellitus (GDM) is known to be associated with pregnancy complications but there is limited evidence about the strength of these associations in recent clinical practice, especially after the introduction of strict guidelines for the management of pregnancies with GDM in a multidisciplinary team setting. The objectives of our study were to first compare the rates of complications in pregnancies with GDM with those that had pre-existing diabetes mellitus and those without diabetes; and second, to derive measures of effect size expressed as odds ratios after adjustment for confounding factors to assess the independent association of GDM in prediction of these pregnancy complications. *Materials and Methods*: This was a prospective cohort study undertaken at a large maternity unit in the United Kingdom between January 2010 and June 2022. We included singleton pregnancies that were booked at our unit at 11–13 weeks’ gestation. Multivariate regression analysis was carried out to determine the risks of complications in pregnancies with GDM after adjusting for pregnancy characteristics. Risks were expressed as odds ratio (OR) (95% confidence intervals [CI]) and expressed graphically in forest plots. *Results*: The study population included 53,649 singleton pregnancies including 509 (1%) with pre-existing DM, 2089 (4%) with GDM and 49,122 (95%) pregnancies without diabetes. Multivariate regression analysis demonstrated that there was a significant independent contribution from GDM in the prediction of adverse outcomes, including maternal complications such as preterm delivery, polyhydramnios, preeclampsia and delivery of large for gestational age neonates and elective caesarean section (CS); and neonatal complications including admission to neonatal intensive care unit, hypoglycaemia, jaundice and respiratory distress syndrome. *Conclusions*: GDM is associated with an increased rate of pregnancy complications compared to those without diabetes, even after adjustment for maternal and pregnancy characteristics. GDM does not increase the risk of stillbirth, hypoxic ischaemic encephalopathy or neonatal death.

## 1. Introduction

Gestational Diabetes Mellitus (GDM) is characterised by impaired glucose tolerance resulting in dysglycaemia with onset or first recognition during pregnancy and resolution following childbirth [1,2]. There is considerable evidence to suggest that maternal hyperglycaemia is associated with adverse pregnancy outcomes, including antenatal, intrapartum and neonatal complications [1,2,3]. The severity of adverse outcomes depends on the degree of hyperglycaemia and the length of fetal exposure to increased glucose levels across the different trimesters of pregnancy [3]. Pre-existing DM is potentially associated with hyperglycaemia not just in the peri-conceptional period but also antenatally; the degree of hyperglycaemia and the associated adverse outcomes in both periods are dependent on how robust the treatment plan is for maintaining glycaemic control. There is considerable evidence that pregnancies with types 1 and 2 DM are associated with an increased chance of maternal and neonatal complications; in particular, there is a substantially increased risk of hypoxic perinatal complications including hypoxic ischaemic encephalopathy (HIE), stillbirths and neonatal death [4,5,6,7]. GDM is also associated with an increased risk of adverse outcomes such as pre-eclampsia, preterm birth, CS, macrosomia and neonatal hypoglycaemia but in contrast to pregnancies with pre-existing DM, there is no evidence of a significant increase in the risk of stillbirth [8,9,10]. Due to the association of pregnancy complications in pregnancies with diabetes, both pre-existing and gestational, the National Institute of Clinical Excellence (NICE) recommended in 2008 that the management of pregnancies with DM should be in a multidisciplinary team (MDT) setting [2]. However, many of the studies reporting results in pregnancies with GDM are from settings before MDT management was the standard of care and, therefore, it is uncertain whether the introduction of MDT care has mitigated the association with pregnancy complications.

The objectives of our study were, first, to determine the absolute risks (AR) of maternal and neonatal complications in pregnancies with GDM compared to those with and without pre-existing DM and, second, to derive accurate estimates of these risks based on odds ratios (OR) calculated after adjustment for confounding pregnancy characteristics to assess the independent association of GDM in the prediction of these pregnancy complications.

## 2. Material and Methods

### 2.1. Study Population

This was an observational cohort study in a large, unselected population of pregnancies undertaken at the Medway Fetal and Maternal Medicine Centre (MFMC), United Kingdom during the study period January 2010 and June 2022. All women booked for their pregnancy care at our hospital attend a Fetal Medicine scan appointment at 11–13 weeks’ gestation for dating of the pregnancy, combined screening for fetal aneuploidies and systematic examination of the fetal anatomy [11,12,13]. The next scan is at 20–22 weeks’ gestation for the examination of fetal growth and anatomy, placenta and umbilical cord and uterine artery Doppler to assess impedance to blood flow. All women have a structured antenatal care plan depending on the presence or absence of risk factors. Those with risk factors are offered an appointment in specific high-risk clinics whereas those without any antenatal risk factors are offered care in the community. We recorded details about maternal demographics, medical and obstetric history, ultrasound scan findings and delivery outcomes in an electronic database (Viewpoint version 5.6; GE Healthcare, Buckinghamshire, UK). Similarly, the outcome data for neonatal care were recorded on the BadgerNet Database (Clevermed Ltd., Edinburgh UK). The protocol for this study was approved by the National Research Ethics Committee (REC reference number 20/HRA/3076).

### 2.2. Inclusion and Exclusion Criteria

We included singleton pregnancies that were booked at our hospital for their pregnancy care prior to 14 weeks’ gestation, those that were managed in an MDT setting by the diabetes team (MDT) and women for whom we have delivery and neonatal data available. The exclusion criteria were multiple pregnancies and those that were lost to follow-up. The pregnancies meeting inclusion criteria were subdivided into those that were diagnosed with GDM, those that had pre-existing DM and those that were non-diabetic.

### 2.3. Screening and Management of Pregnancies with Gestational Diabetes Mellitus

Screening for pregnancies at risk of GDM in our hospital is based on specific risk factors from maternal demographics, previous obstetric history, family history and findings from the current pregnancy in line with recommendations from NICE [2]. Maternal factors include a body mass index (BMI) of >30 kg/m^2^ and a non-white ethnic origin; previous obstetric risk factors include those who delivered a macrosomic neonate with birthweight (BW) >4500 g (g), those with a prior diagnosis of GDM, family history of a first-degree relative with a diagnosis of DM and findings from the current pregnancy including a large for gestational age (LGA) fetus, polyhydramnios on ultrasound scan, the presence of glycosuria on a urinary dipstick test in pregnancy (1+ on two occasions or 2+ on one occasion) and maternal intake of anti-psychotic drugs such as quetiapine, risperidone, clozapine and olanzapine. In all pregnancies with these risk factors, an oral glucose tolerance test (OGTT) was carried out with the administration of a 75 g glucose challenge, and a diagnosis of GDM was made if the fasting blood glucose level was ≥5.6 mmol/L or the 2 h blood glucose level was ≥7.8 mmol/L [2]. The gestational age for OGTT testing depended on the indication; in those with a previous pregnancy with GDM, an OGTT was performed in the first trimester and repeated at 24–28 weeks if normal in the first trimester; in those with maternal demographic risk factors or obstetric and family history risk factors, the testing was performed at 24–28 weeks; and in those with current pregnancy risk factors, testing was performed when a diagnosis of either a LGA foetus or polyhydramnios was made. During the pandemic, diagnosis of GDM was also based on testing for HbA1C and random or fasting plasma glucose (RPG or FPG, respectively). GDM was diagnosed from either an HbA1c, a random plasma glucose (RPG) test or a fasting plasma glucose (FPG) test, and parameters for diagnosis were dependent on the gestational age at testing. GDM at the pregnancy booking appointment was diagnosed when an HbA1c was between 41–47/mmol/mol or when an RPG was between 9–11 mmol/L. Screening for GDM was repeated at 28 weeks for all pregnancies with risk factors, and a diagnosis was made with an HbA1c ≥ 39 mmol/mol or an FPG ≥ 5.3 mmol/L.

All pregnancies with a diagnosis of GDM were managed by a diabetes MDT in a specialist clinic; the team included an obstetrician, a diabetologist, a dietician and specialist midwives. The appointments in these specialist clinics included those for the assessment of foetal well-being and those for the management of maternal diabetes and euglycaemia. Fetal well-being was assessed in a dedicated diabetes clinic in the Foetal Medicine Unit, which was held on the same day as the multidisciplinary antenatal clinic. All pregnancies with GDM had routine scan appointments at 11–13 weeks for pregnancy dating and screening for aneuploidies and at 20–22 weeks for the assessment of foetal growth and anatomy, amniotic fluid, placental location and assessment of impedance to blood flow in uterine arteries by Doppler ultrasound. All pregnancies diagnosed with GDM were offered ultrasound scans at 28 weeks and every 3–4 weeks thereafter until delivery to assess foetal growth, amniotic fluid and Doppler evaluation of foetal-placental circulation in the specialist diabetes clinic. Appointments for maternal diabetes included regular appointments in the diabetes clinic for the assessment of glycaemic control and management of associated pregnancy and medical complications. All mothers were provided advice about diet and exercise by a specialist dietician and nutrition specialist and taught to self-monitor capillary blood glucose levels to maintain a target of <5.3 mmol/L at fasting, <7.8 mmol/L 1-h post meals, and <6.4 mmol/L 2-h post meals. Pregnancies with fasting blood glucose levels of <7.0 mmol/L are offered a 2-week trial of diet modification and exercise to assess their glycaemic response to this intervention; if the blood glucose is not below target levels, then they are advised to commence treatment with metformin. Treatment with insulin is advised if the fasting blood glucose levels are ≥7.0 mmol/L, there are contraindications to metformin or there is no satisfactory response to metformin. Women are advised to consider elective delivery around 37–38 completed weeks’ gestation if they are on treatment with insulin or by 40 completed weeks’ gestation if they are controlled on diet alone.

### 2.4. Outcome Measures

We assessed maternal and neonatal adverse outcomes. Maternal adverse outcomes included congenital defects, miscarriage and stillbirth, preterm birth, foetal growth abnormalities, polyhydramnios and hypertensive disorders, whereas the neonatal adverse outcomes included admission to the neonatal intensive care unit (NICU), hypoxic ischaemic encephalopathy (HIE), hypoglycaemia, respiratory distress syndrome (RDS), jaundice and neonatal death. Congenital defects included those related to the central nervous system, cardiovascular, renal, gastrointestinal, musculoskeletal and genetic causes. Miscarriage and stillbirth were defined as fetal death prior to and at or after 24 weeks’ gestation, respectively. Fetal growth abnormalities were divided into those that were small (<10th percentile) or those that were large (>90th percentile) for gestational age. (SGA and LGA, respectively) [14]. Polyhydramnios was defined as the deepest pool > 8 cm; these were further divided into those that were mild (8–11 cm) and moderate/severe (≥12 cm) [15]. Gestational hypertension was defined when maternal blood pressure (BP) was >140/90 mm Hg at or after 20 weeks’ gestation without significant proteinuria. Preeclampsia was defined as the presence of gestational hypertension with significant proteinuria (protein-creatinine ratio of >30 mg/mmol) [16]. Caesarean sections were classified as elective (planned) or emergency [17]. An estimated blood loss (EBL) > 1000 mL in the third stage of labour was classified as post-partum haemorrhage, which was further divided into moderate (1001–2000 mL) and severe (> 2000 mL) [18]. Third- and fourth-degree tears involving an injury to the anal sphincter complex and anorectal mucosa were categorized as obstetric anal sphincter injury (OASIS) [19]. Vaginal delivery that required additional manoeuvres for the delivery of the fetal body after delivery of the head was classified as shoulder dystocia [20]. HIE was defined as the presence of an abnormal neurologic function secondary to perinatal hypoxia reflected in either a 5-min APGAR score < 5 or umbilical artery cord pH < 7.0 or base deficit > 12 mmol/L, supported by neuroimaging evidence of acute brain injury [21]. Hypoglycaemia was defined by neonatal serum glucose levels of <2.6 mmol/L [22]. Diagnosis of neonatal jaundice was based on visual observation of yellow discolouration of the skin or sclera with an elevated serum bilirubin measurement. RDS was defined as the inability to maintain adequate oxygen saturations with spontaneous respirations and the need for additional respiratory support.

### 2.5. Statistical Analysis

Maternal and pregnancy characteristics were compared between those without diabetes and those who had GDM and pre-existing DM. We used the χ2-square test or Fisher’s exact test for categorical variables and the Kruskal–Wallis and Mann–Whitney U-test for continuous variables, respectively. Significance was assumed at 5%. Post hoc Bonferroni correction was made to adjust the significance level for multiple comparisons to avoid a type I error.

Rates of maternal and neonatal complications in pregnancies with GDM, pre-existing DM and non-diabetic controls were used to calculate absolute risks (AR,%). We calculated the unadjusted odds ratio (OR) with 95% CI from univariable logistic regression analysis. In the case of each adverse outcome, multivariable logistic regression analysis with backwards stepwise elimination was then carried out to estimate the adjusted OR (95%CI). Forest plots were constructed to graphically express the risk of maternal and neonatal complications in pregnancies with GDM and pre-existing DM compared to non-diabetic controls. The data for pre-existing DM were published in a previous publication [4]. The statistical package SPSS 24.0 (IBM SPSS Statistics for Windows, Version 24.0, Armonk, NY, USA: IBM Corp; 2016) and MedCalc Statistical Software version 18.5 (MedCalc Software, Ostend, Belgium, 2018) were used for data analyses.

## 3. Results

### 3.1. Study Population

The study population included 53,649 singleton pregnancies; we excluded 3005 (5.6%) pregnancies, including 1929 pregnancies (3.6%) who were lost to follow-up, 581 (1.1%) who had a miscarriage and 495 (0.9%) that had a termination of pregnancy. The final study population was thus formed of 50,644 singleton pregnancies including 509 (1%) with pre-existing DM, 2089 (4.0%) with GDM and 49,122 (95%) controls without diabetes.

### 3.2. Maternal and Pregnancy Characteristics

The maternal and pregnancy characteristics in the study population are shown in Table 1.

#### 3.2.1. Gestational Diabetes Mellitus vs. Non-Diabetic Pregnancies

In pregnancies with GDM compared to non-diabetic controls, the median maternal age (31.6 vs. 29.0 years; *p* < 0.01), weight (82.0 vs. 68.6 kg; *p* < 0.01), BMI (30.8 vs. 25.2 kg/m^2^; *p* < 0.01) and BW percentile (68.5 vs. 52.4; *p* < 0.01) were higher, whereas maternal height (163 vs. 165 cm; *p* < 0.01) and gestational age at delivery (38.3 vs. 39.6 weeks; *p* < 0.01) were lower. In pregnancies with GDM compared to those without, there was a higher prevalence of obesity with BMI > 35 and 40 (28.0 vs. 9.3%; *p* < 0.01 and 11.2 vs. 3.2%; *p* < 0.01, respectively), women of Afro-Caribbean racial origin (7.4 vs. 3.1%; *p* < 0.01), South Asian racial origin (12.7 vs. 4.1%; *p* < 0.001), East Asian racial origin (1.1 vs. 0.4%; *p* < 0.01), conception by in vitro fertilisation (2.9 vs. 1.6%; *p* < 0.01) and maternal chronic hypertension (2.7 vs. 1.0%; *p* < 0.01), whereas there were fewer women of Caucasian origin (67.6 vs. 91.2%; *p* < 0.01) and cigarette smokers (10.1 vs. 15.4%; *p* < 0.01) (Table 1).

#### 3.2.2. Pre-Existing Diabetes Mellitus vs. Non-Diabetic Pregnancies

Pregnancies with pre-existing DM compared to those without diabetes had a higher median maternal age (30.3 vs. 29.0%; *p* < 0.01), weight (82.0 vs. 68.6 kg; *p* < 0.01), BMI (30.2 vs. 25.2 kg/m^2^; *p* < 0.01) and BW percentile (82.8 vs. 52.4; *p* < 0.01) whereas gestational age at delivery was lower (37.1 vs. 39.5 weeks; *p* < 0.01). Pregnancies with pre-gestational DM had a higher prevalence of BMI > 35 and >40 (30.8 vs. 9.3%; *p* < 0.01 and 15.1 vs. 3.2%; *p* < 0.01, respectively), women of Afro-Caribbean racial origin (5.5 vs. 3.1%; *p* < 0.01), South Asian racial origin (7.9 vs. 4.1%; *p* < 0.001), cigarette smokers (22.4 vs. 15.4%; *p* < 0.01), pregnancies conceived by IVF (2.8 vs. 1.6%; *p* < 0.01) and those with chronic hypertension (5.3 vs. 1.0%; *p* < 0.01), epilepsy (1.8 vs. 0.8%; *p* < 0.01) and autoimmune disorders (36.3 vs. 1.0%; *p* < 0.01) (Table 1).

#### 3.2.3. Gestational Diabetes Mellitus vs. Pre-Existing Diabetes Mellitus

In pregnancies with GDM compared to pre-existing DM, the median maternal age (31.6 vs. 30.3%; *p* < 0.0167) and gestational age at delivery (38.3 vs. 37.1 weeks; *p* < 0.0167) were higher, whereas birthweight percentile was lower (68.5 vs. 82.8; *p* < 0.0167). The prevalence of women with South Asian racial origin was substantially higher in pregnancies with GDM compared to those with pre-existing DM (12.7 vs. 7.9%; *p* < 0.0167) but there was a lower prevalence of pregnancies with a BMI > 40 (11.2 vs. 15.1 kg/m^2^; *p* < 0.0167), those that were cigarette smokers (10.1 vs. 22.4%; *p* < 0.0167), those with chronic hypertension (2.7 vs. 5.3%; *p* < 0.0167) and pregnancies with autoimmune disorders (1.3 vs. 36.3%; *p* < 0.0167) (Table 1).

### 3.3. Antenatal Adverse Outcomes

#### 3.3.1. Gestational Diabetes Mellitus vs. Non-Diabetic Pregnancies

In pregnancies with GDM compared to those without diabetes, there was an increased rate of preterm delivery <37 weeks (10.9 vs. 6.1%; *p* < 0.01), delivery of LGA neonate (25.3 vs. 10.7%; *p* < 0.01), mild (6.1 vs. 1.9%; *p* < 0.01) and severe (0.4 vs. 0.1%; *p* < 0.01) polyhydramnios, gestational hypertension (2.7 vs. 1.6; *p* < 0.01) and preeclampsia (4.1 vs. 2.3%; *p* < 0.01), whereas the rate of delivery of SGA neonates was lower (6.7 vs. 10.9%; *p* < 0.01). There were no differences in fetal defects (*p* = 0.133), miscarriage (*p* = 0.111) or stillbirth (*p* = 0.551) (Table 2).

**Figure 1:**
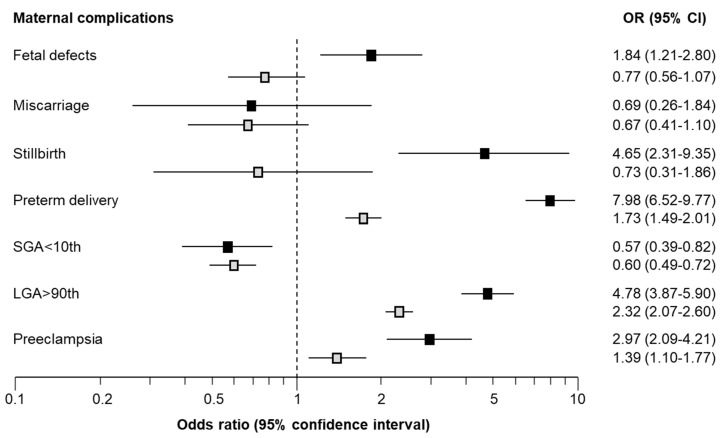
Forest plot with odds ratios (OR) with 95% confidence intervals (CI) demonstrating the association of maternal complications in those with gestational diabetes mellitus (GDM) (grey squares) and pre-existing DM (black squares) compared to pregnancies without DM.

Multivariable logistic regression analysis demonstrated that pregnancies with GDM remained at an increased risk of preterm delivery <37 weeks (OR = 1.73; 95%CI: 1.49–2.01), delivery of LGA neonate (OR = 2.32; 95%CI: 2.07–2.60), mild polyhydramnios (OR = 2.16; 95%CI: 1.76–2.65) and preeclampsia (OR = 1.39; 95%CI: 1.10–1.77) but there was no significant increase in the risk of severe polyhydramnios (*p* = 0.281) or gestational hypertension (*p* = 0.384) (Table 3, Figure 1).

#### 3.3.2. Pre-Existing Diabetes Mellitus vs. Non-Diabetic Pregnancies

Pregnancies with pre-existing DM compared to non-diabetic controls had a higher prevalence of CNS and CVS defects (1.0 vs. 0.3%; *p* = 0.018 and 2.6 vs. 0.6%; *p* < 0.01, respectively) but there was no difference in the prevalence of renal, musculoskeletal, gastrointestinal or genetic defects. In pregnancies with DM compared to those without, there was no significant difference in the rate of miscarriage (*p* = 0.454), whereas there was a substantially increased rate of stillbirth (1.8% vs. 0.3%; *p* < 0.01). The risk of preterm delivery <32 weeks (3.9 vs. 0.9%; *p* < 0.01) and <37 weeks (36.1 vs. 6.1%; *p* < 0.01), mild and moderate/severe polyhydramnios (12.8 vs. 1.9%; *p* < 0.01 and 1.6 vs. 0.1%; *p* < 0.01, respectively), preeclampsia (8.4 vs. 2.3%; *p* < 0.01) and delivery of LGA neonates (39.3 vs. 10.7%; *p* < 0.01) was significantly increased in those with pre-existing DM compared to non-diabetic controls but there was no difference in the rate of gestational hypertension (*p* = 0.531) (Table 2).

Multivariable logistic regression analysis demonstrated that pregnancies with pre-existing DM remained at an increased risk of having a pregnancy affected by defects of the central nervous system (OR = 3.42; 95%CI: 1.39–8.41), cardiovascular system (OR = 4.41; 95%CI: 2.50–7.78), stillbirth (OR = 4.65; 95%CI: 2.31–9.35), preterm delivery <32 and 37 weeks (OR = 4.31; 95%CI: 2.65–7.01 and OR = 7.98; 95%CI: 6.52–9.77, respectively), delivery of LGA neonate (OR = 4.78; 95%CI: 3.87–5.90), mild and moderate/severe polyhydramnios (OR = 3.99; 95%CI: 2.97–5.36 and OR = 6.75; 95%CI: 3.15–14.47, respectively) and preeclampsia (OR = 2.97; 95%CI: 2.09–4.21) but there was no significant increase in the risk of gestational hypertension (*p* = 0.384) (Supplementary Table S1, Figure 1).

#### 3.3.3. Gestational Diabetes Mellitus vs. Pre-Existing Diabetes Mellitus

In pregnancies with GDM compared to pre-existing DM, there is a lower prevalence of foetal defects associated with the central nervous and cardiovascular systems (0.1 vs. 1.9%; *p* < 0.0167; 0.3 vs. 2.6%; *p* > 0.001, respectively), stillbirths (0.2 vs. 1.8%; *p* < 0.001), preterm delivery before 32 and 37 weeks (1.0 vs. 3.9%; *p* < 0.0001; 10.9 vs. 36.1%; *p* > 0.001, respectively), polyhydramnios—both mild and moderate/severe (6.1 vs. 12.8%; *p* < 0.001; 0.4 vs. 1.6%; *p* > 0.001, respectively)—the delivery of LGA neonates (25.3 vs. 39.3%; *p* < 0.001) and preeclampsia (4.1 vs. 8.4%; *p* < 0.001) (Table 2).

### 3.4. Intrapartum Adverse Outcomes

#### 3.4.1. Gestational Diabetes Mellitus vs. Non-Diabetic Controls

In pregnancies with GDM compared to non-diabetic controls, there was a significantly increased rate of IOL (45.2 vs. 26.7%; *p* < 0.01), elective and emergency CS (25.1 vs. 11.7%; *p* < 0.01 and 23.7 vs. 16.9%; *p* < 0.01, respectively) and moderate PPH (10.1 vs. 7.3%; *p* < 0.01), whereas the rate of unassisted vaginal delivery was lower (43.9 vs. 62.7; *p* < 0.01). There was no significant difference in the rate of operative vaginal deliveries (*p* = 0.028), severe PPH (*p* = 0.166), OASIS (*p* = 0.298) or shoulder dystocia (*p* = 0.348) (Table 4).

Multivariable logistic regression analysis demonstrated that there was an increased risk of IOL (OR = 2.09; 95%CI: 1.91–2.29) and elective CS (OR = 1.68; 95%: 1.51–1.88) but there was no significant independent association with the risk of moderate PPH (*p* = 0.118) or emergency CS (*p* = 0.603) (Table 5).

#### 3.4.2. Pre-Existing Diabetes Mellitus vs. Non-Diabetic Controls

In pregnancies with pre-existing DM compared to those without, there was a significantly lower rate of unassisted (27.9 vs. 62.7%; *p* < 0.01) and operative vaginal births (4.5 vs. 8.7%; *p* < 0.01), increased rate of elective and emergency CS (35.0 vs. 11.7%; *p* < 0.0167 and 32.6 vs. 16.9%; *p* < 0.0167, respectively) and moderate PPH (13.0 vs. 7.3%; *p* < 0.01) but no significant difference in severe PPH, OASIS or shoulder dystocia (Table 4).

Multivariable logistic regression analysis demonstrated that after adjusting for maternal and pregnancy characteristics, the risk of elective and emergency CS (OR = 2.62; 95%: 2.12–3.23 and OR = 1.46; 95%CI: 1.17–1.82, respectively) was increased but there was no significant independent association with PPH (Supplementary Table S2).

#### 3.4.3. Gestational Diabetes Mellitus vs. Pre-Existing Diabetes Mellitus

In pregnancies with GDM compared to pre-existing DM, there was a significantly higher rate of IOL (45.2 vs. 37.7%; *p* < 0.0167) and unassisted spontaneous vaginal deliveries (43.9 vs. 27.9%; *p* < 0.001) but a lower rate of elective and emergency CS (25.1 vs. 35.0%; *p* < 0.001 and 23.7 vs. 32.6%%; *p* < 0.001, respectively) (Table 4).

### 3.5. Neonatal Adverse Outcomes

#### 3.5.1. Gestational Diabetes Mellitus vs. Non-Diabetic Controls

In pregnancies with GDM compared to non-diabetic controls, there is a significantly increased risk of neonatal complications with a higher incidence of admission to NICU (25.8 vs. 14.5%; *p* < 0.01), hypoglycaemia (4.5 vs. 1.0%; *p* < 0.01), jaundice (11.6 vs. 5.2%; *p* < 0.01) and RDS (6.5 vs. 3.3%; *p* < 0.01), whereas there was no significant difference in the rate of HIE (*p* = 0.639) (Table 6).

**Figure 2:**
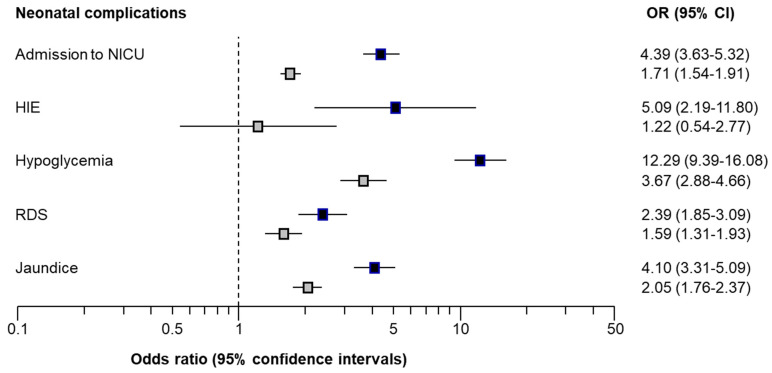
Forest plot with odds ratios (OR) with 95% confidence intervals (CI) demonstrating the association of neonatal complications in those with gestational diabetes mellitus (GDM) (grey squares) and pre-existing DM (black squares) compared to pregnancies without DM.

Multivariable logistic regression analysis demonstrated that in pregnancies with GDM, the association with neonatal complications remained even after adjusting for maternal and pregnancy characteristics with a significantly increased risk of admission to NICU (OR = 1.71; 95%CI: 1.54–1.91), hypoglycaemia (OR = 3.67; 95%CI: 2.88–4.66), jaundice (OR = 2.05; 95%CI: 1.76–2.37) and RDS (OR = 1.59; 95%CI: 1.31–1.93) (Table 7, Figure 2).

#### 3.5.2. Pre-Existing Diabetes Mellitus vs. Non-Diabetic Controls

In pregnancies with pre-existing DM compared to those without, there was a higher risk of admission to NICU (52.5 vs. 14.5%; *p* < 0.01), HIE (1.2 vs. 0.2%; *p* < 0.01), hypoglycaemia (20.2 vs. 1.0%; *p* < 0.01), jaundice (26.3 vs. 5.2%; *p* < 0.01), RDS (17.3 vs. 3.3%; *p* < 0.01) and rate of neonatal death (0.4 vs. 0.1%; *p* < 0.01) (Table 6).

Multivariate logistic regression analysis demonstrated that in pregnancies with pre-existing DM, the association with neonatal complications remained even after adjusting for maternal and pregnancy characteristics with a significantly increased risk of admission to NICU (OR = 4.39; 95%CI: 3.63–5.32), HIE (OR = 5.09; 95%CI 2.19–11.80), hypoglycaemia (OR = 12.29; 95%CI: 9.39–16.08), jaundice (OR = 4.10; 95%CI: 3.31–5.09) and RDS (OR = 2.39; 95%CI: 1.85–3.09) (Supplementary Table S3, Figure 2).

#### 3.5.3. Gestational Diabetes Mellitus vs. Pre-Existing Diabetes Mellitus

In pregnancies with GDM compared to pre-existing DM, there was a substantially reduced risk of neonatal complications. There was a lower risk of admission to NICU (25.8 vs. 52.5%; *p* < 0.001), hypoglycaemia (4.5 vs. 20.2%; *p* < 0.001), jaundice (11.6 vs. 26.3%; *p* < 0.001), RDS (6.5 vs. 17.3%; *p* < 0.001), HIE (0.3 vs. 1.2%; *p* < 0.0167) and neonatal death (0.0 vs. 0.4%; *p* < 0.0167) (Table 6).

## 4. Discussion

### 4.1. Principal Findings of the Study

These findings confirm that, first, peri-conceptional and first-trimester hyperglycaemia is associated with pregnancy complications such as increased association with congenital foetal defects in pre-existing DM but not in those with GDM; second, the higher risks of antenatal, intrapartum and neonatal complications in pregnancies with pre-existing DM and GDM reflect consequences of second- and third-trimester hyperglycaemia, which, despite treatment in MDT clinics, remains a significant challenge; third, the effect size noted in pregnancies with pre-existing DM and GDM compared to non-diabetic controls is likely to be due to the severity of hyperglycaemia in those with pre-existing disease compared to those with GDM and fourth, and there is a substantially increased risk of hypoxic perinatal morbidity and mortality in those with pre-existing DM compared to non-diabetic controls, reflected in HIE and stillbirth, whereas in pregnancies with GDM, there is no significant difference in the risk of these complications, the rates of which are similar to non-diabetic controls.

### 4.2. Strengths and Limitations

The strengths of the study are, first, the examination of a large cohort of pregnancies consecutively screened and delivered in a large tertiary referral foetal medicine, obstetric and neonatal unit; second, the inclusion of pregnancies managed in a specialist multidisciplinary high-risk clinic by a specialist obstetrician, endocrinologist and specialist midwife; third, the review of case notes of all pregnancies with pre-existing DM and ascertainment of maternal and neonatal adverse outcomes by reviewing maternity records to ensure the accuracy of pregnancy outcomes and, fourth, the use of multivariable regression analysis to adjust for confounding factors in maternal and pregnancy characteristics to derive adjusted measures of effect size for associations of pre-existing DM and GDM with adverse outcomes. A limitation of our study is that this is a single-centre study, and the reported incidence of maternal and neonatal complications is a consequence of contemporary obstetric care provided in multidisciplinary obstetric high-risk clinics and is likely to be affected by the characteristics of our population and the protocols for antenatal, intrapartum and neonatal care in the United Kingdom. However, although there are variations in clinical care between different settings, the absolute and adjusted risks of complications in pregnancies with pre-existing DM compared to those without diabetes are likely to be accurate estimates, given a similar multidisciplinary setting as described in our study and clinical care based on standardised protocols for multidisciplinary care. Our study was limited to singleton pregnancies, and the estimates of risk in multiple pregnancies may be higher.

### 4.3. Comparison with Other Studies

The results of our study demonstrate that despite the management of pregnancies with diabetes in an MDT setting, there remains a substantially high rate of maternal and neonatal complications in these high-risk pregnancies [23,24,25,26]. In our study, the rate of preeclampsia in mothers with pre-existing DM was 4–5-fold higher, and in pregnancies with GDM, was 2-fold higher compared to pregnancies without diabetes. These findings are similar to those from a large population study including over 270,000 pregnancies in which the authors reported that the rates of preeclampsia in those with GDM were almost double that of non-diabetic mothers [23]. Another study in pregnancies with GDM reported that the rate of preeclampsia was almost double (OR 1.81), compared to the non-diabetic population, even after adjustment for maternal factors and medical history [24]. In our study, the rate of delivery of LGA neonates was significantly higher in pre-existing DM and GDM compared to those without diabetes (39.3% and 25.3% vs. 10.9%, respectively). A similar increased risk of delivery of LGA neonates was also noted in a large study from Canada in which the authors reported that in pregnancies with pre-existing DM and GDM, the adjusted OR was 3.73 and 1.63, respectively [25]. Our study noted an increased association with delivery by CS in both pre-existing and gestational DM compared to non-diabetic controls. In pregnancies with pre-existing DM, there was a 2.5- and 1.5-fold increase in the risk of elective and emergency CS compared to non-diabetics, whereas in those with GDM, there was a 1.7-fold increase in the rate of elective CS but no significance difference in risk of emergency CS, compared to non-diabetic controls when other confounding factors were adjusted for. Similar results were also noted in other studies, which reported an increased risk of CS in both pre-existing and GDM [24,25]. Lai et al. reported a 2.5-fold and 1.5-fold increase in the risk of CS in 2485 pregnancies with pre-existing DM and 18,137 with GDM, compared to 306,576 pregnancies without DM [25]. In our study, there was a 4–5-fold increase in the risk of stillbirths in pregnancies with pre-existing DM compared to those without diabetes, whereas in those with GDM, there was no significant difference in the rate of stillbirths compared to non-diabetic controls. This is similar to data from other studies, which also reported that there is a significantly increased association with stillbirths in pre-existing DM but not in those with GDM [24,25,26].

## 5. Conclusions

The findings of our study demonstrate that despite the provision of multidisciplinary care in specialist clinics, there remains an increased risk of pregnancy complications including the risk of perinatal morbidity and mortality in pregnancies with diabetes mellitus compared to non-diabetic controls. In pregnancies with pre-existing DM, there is an increased risk of antenatal adverse outcomes beginning from the first trimester of pregnancy through until delivery, intrapartum and neonatal complications. In pregnancies with GDM, the main increase is in the risk of complications in the third trimester of pregnancy such as preterm delivery and preeclampsia, as well as an increased association with delivery by CS and neonatal complications. The rate of complications is significantly higher in those with pre-existing DM compared to GDM, which is likely to reflect the severity of hyperglycaemia. Further research is needed to investigate different pathways and strategies to improve control of maternal glycaemia in pregnancies with diabetes to reduce the risk of complications and prevent hypoxic perinatal adverse outcomes such as HIE, stillbirths and neonatal deaths, which unfortunately remain the same as they were decades ago.

## Figures and Tables

**Table 1:** Maternal demographic and pregnancy characteristics in pregnancies with gestational diabetes mellitus (GDM), those with pre-existing DM compared to those without diabetes.

Maternal Antenatal Adverse Outcomes	Non-Diabetes (*n* = 49,122)	Pre-Existing DM (*n* = 509)	GDM (*n* = 2089)
Maternal age in years, median (IQR)	29.0 (25.0–32.9)	30.3 (26.2–34.7) **	31.6 (27.9–35.3) **†
Maternal weight in kg, median (IQR)	68.6 (59.5–81.0)	82.0 (70.0–98.0) **	82.0 (68.5–97.0) **
Maternal height in cm, median (IQR)	165 (160–169)	164 (160–168)	163 (159–168) **
Maternal BMI in kg/m^2^, median (IQR)	25.2 (22.2–29.6)	30.2 (26.0–36.5) **	30.8 (26.2–35.8) **
>35, n (%)	4592 (9.3)	157 (30.8) **	584 (28.0) **
>40, n (%)	1564 (3.2)	77 (15.1) **	233 (11.2) **†
Racial origin			
Caucasian, n (%)	44,819 (91.2)	430 (84.5) **	1412 (67.6) **
Afro-Caribbean, n (%)	1519 (3.1)	28 (5.5) *	155 (7.4) **
South Asian, n (%)	2004 (4.1)	40 (7.9) **	265 (12.7) **†
East Asian, n (%)	205 (0.4)	2 (0.4)	23 (1.1) **
Mixed, n (%)	575 (1.2)	9 (1.8)	30 (1.4)
Conception			
Spontaneous, n (%)	48,357 (98.4)	495 (97.2)	2029 (97.1)
In vitro fertilisation, n (%)	765 (1.6)	14 (2.8) *	60 (2.9) *
Cigarette smoking, n (%)	7575 (15.4)	114 (22.4) **	212 (10.1) **†
History of medical disorders			
Chronic hypertension, n (%)	502 (1.0)	27 (5.3) **	57 (2.7) **†
Bronchial asthma, (%)	3079 (6.3)	35 (6.9)	108 (5.2)
Epilepsy, n (%)	410 (0.8)	9 (1.8) *	14 (0.7)
Thyroid disorders, n (%)	579 (1.2)	10 (2.0)	47 (2.2)
Autoimmune disorders, n (%)	506 (1.0)	185 (36.3) **	28 (1.3) †
GA at delivery, median (IQR)	39.5 (38.6–40.5)	37.1 (36.0–38.1) **	38.3 (37.5–39.3) **†
BW in Kg, median (IQR)	3.42 (3.07–3.75)	3.31 (2.91–3.67) **	3.42 (3.08–3.77) †
BW percentile, median (IQR)	52.4 (25.5–77.1)	82.8 (52.4–96.8) **	68.5 (38.9–90.4) **†

IQR = interquartile range; DM = Diabetes mellitus; GA = Gestational age; BW = Birthweight; Significance level p * and † *p* < 0.0167; ** *p* < 0.001. Post hoc Bonferroni correction made for multiple comparisons.; * Comparison of pre-existing DM and GDM with non-diabetic pregnancies; † Comparison between GDM and pre-existing DM.

**Table 2:** Absolute risk of maternal antenatal complications in pregnancies with gestational diabetes mellitus (GDM) compared to those with pre-existing DM and those without diabetes.

Antenatal Adverse Outcomes	Non-Diabetes (*n* = 49,122)	Pre-Existing DM (*n* = 509)	GDM (*n* = 2089)
Fetal defects	1181 (2.4)	22 (4.3) **	39 (1.9)
Central nervous system, n (%)	145 (0.3)	5 (1.0) *	3 (0.1) †
Cardiovascular system, n (%)	271 (0.6)	13 (2.6) **	6 (0.3) ‡
Renal, n (%)	308 (0.6)	3 (0.6)	15 (0.7)
Gastrointestinal, n (%)	127 (0.3)	0	3 (0.1)
Musculoskeletal, n (%)	114 (0.2)	1 (0.2)	7 (0.3)
Genetic, n (%)	216 (0.4)	0	5 (0.2)
Fetal death			
Miscarriage, n (%)	561 (1.1)	4 (0.8)	16 (0.8)
Stillbirth, n (%)	154 (0.3)	9 (1.8) **	5 (0.2) ‡
Preterm delivery			
<32 weeks, n (%)	453 (0.9)	20 (3.9) **	21 (1.0) ‡
<37 weeks, n (%)	3015 (6.1)	184 (36.1) **	227 (10.9) **‡
Fetal growth abnormalities			
SGA <10th percentile, n (%)	5370 (10.9)	35 (6.9) *	140 (6.7) **
LGA >90th percentile, n (%)	5262 (10.7)	200 (39.3) **	529 (25.3) **‡
Polyhydramnios			
Mild, n (%)	922 (1.9)	65 (12.8) **	128 (6.1) **‡
Moderate/severe, n (%)	68 (0.1)	8 (1.6) **	8 (0.4) **‡
Obstetric complications			
Gestational hypertension, n (%)	792 (1.6)	10 (2.0)	57 (2.7) **
Preeclampsia, n (%)	1142 (2.3)	43 (8.4) **	86 (4.1) **‡

SGA = small for gestational age; LGA = large for gestational age; Significance level p * and † *p* < 0.0167; ** and ‡ *p* < 0.001. Post hoc Bonferroni correction made for multiple comparisons. * Comparison of pre-existing DM and GDM with non-diabetic pregnancies; † Comparison between GDM and pre-existing DM.

**Table 3:** Univariable and multivariable logistic regression analysis demonstrating the association of gestational diabetes mellitus (GDM) with antenatal adverse outcomes.

Antenatal Adverse Outcomes	Univariate Analysis		Multivariate Analysis	
	OR (95% CI)	*p* Value	OR (96%CI)	*p* Value
Fetal defects	0.77 (0.56–1.07)	0.463	-	-
Central nervous system	0.49 (0.16–1.53)	0.216	-	-
Cardiovascular	0.52 (0.23–1.17)	0.113	-	-
Renal	1.15 (0.68–1.93)	0.607	-	-
Gastrointestinal	0.56 (0.18–1.75)	0.314	-	-
Musculoskeletal	1.45 (0.67–3.10)	0.345	-	-
Genetic	0.54 (0.22–1.32)	0.178	-	-
Fetal death				
Miscarriage	0.67 (0.41–1.10)	0.113	-	-
Stillbirth	0.73 (0.31–1.86)	0.552	-	-
Preterm delivery				
<32 weeks	1.09 (0.70–1.69)	0.698	-	-
<37 weeks	1.86 (1.62–2.15)	<0.001	1.73 (1.49–2.01)	<0.001
Fetal growth abnormalities				
SGA <10th percentile	0.59 (0.49–0.70)	<0.001	0.60 (0.49–0.72)	<0.001
LGA >90th percentile	2.83 (2.55–3.13)	<0.001	2.32 (2.07–2.60)	<0.001
Polyhydramnios				
Mild	3.42 (2.83–4.14)	<0.001	2.16 (1.76–2.65)	<0.001
Moderate/severe	2.90 (1.39–6.04)	<0.001	-	-
Obstetric complications				
Gestational hypertension	1.71 (1.30–2.25)	<0.001	-	-
Preeclampsia	1.80 (1.44–2.26)	<0.001	1.39 (1.10–1.77)	0.006

OR = odds ratio; CI = confidence interval; SGA = small for gestational age; LGA = large for gestational age.

**Table 4:** Absolute risk of maternal intrapartum adverse events in pregnancies with gestational diabetes mellitus (GDM) compared to those with pre-existing DM and those without diabetes.

Maternal Antenatal Adverse Outcomes	Non-Diabetes (*n* = 49,122)	Pre-Existing DM (*n* = 509)	GDM (*n* = 2089)
Induction of labour, n (%)	13,124 (26.7)	192 (37.7) **	945 (45.2) **†
Mode of delivery			
Unassisted vaginal, n (%)	30,820 (62.7)	142 (27.9) **	917 (43.9) **‡
Operative vaginal, n (%)	4282 (8.7)	23 (4.5) **	153 (7.3)
Elective caesarean section, n (%)	5728 (11.7)	178 (35.0) **	525 (25.1) **‡
Emergency caesarean section, n (%)	8326 (16.9)	166 (32.6) **	496 (23.7) **‡
Failure to progress, n (%)	2796 (5.7)	42 (8.3) *	194 (9.3) **
Fetal distress, n (%)	4164 (8.5)	88 (17.3) **	207 (9.9) *‡
Postpartum haemorrhage, n (%)			
Moderate, n (%)	3607 (7.3)	66 (13.0) **	212 (10.1) **
Severe, n (%)	711 (1.4)	13 (2.6) *	38 (1.8)
Obstetric anal sphincter injury, n (%)	777 (1.6)	7 (1.4)	27 (1.3)
Shoulder dystocia, n (%)	571 (1.2)	4 (0.8)	29 (1.4)

Significance level p * and † *p* < 0.0167; ** and ‡ *p* < 0.001. Post hoc Bonferroni correction made for multiple comparisons. * Comparison of pre-existing DM and GDM with non-diabetic pregnancies; † Comparison between GDM and pre-existing DM.

**Table 5:** Univariable and multivariable logistic regression analysis demonstrating the association of gestational diabetes mellitus (GDM) with intrapartum pregnancy complications.

Intrapartum Adverse Outcomes	Univariate Analysis		Multivariate Analysis	
	OR (95% CI)	*p* Value	OR (96%CI)	*p* Value
Induction of labour	2.26 (2.07–2.48)	<0.001	2.09 (1.91–2.29)	<0.001
Mode of delivery				
Spontaneous vaginal	0.46 (0.43–0.51)	<0.001	0.65 (0.59–0.72)	<0.001
Instrumental deliveries	0.83 (0.70–0.98)	0.027	-	-
Elective caesarean section	2.54 (2.30–2.82)	<0.001	1.68 (1.51–1.88)	<0.001
Emergency caesarean section	1.53 (1.38–1.69)	<0.001	-	-
Failure to progress	1.70 (1.46–1.98)	<0.001	-	-
Fetal distress	1.19 (1.03–1.38)	0.022	-	-
Postpartum haemorrhage				
Moderate	1.43 (1.23–1.65)	<0.001	-	-
Severe	1.26 (0.91–1.75)	0.167	-	-
Obstetric anal sphincter injury	0.82 (0.55–1.20)	0.298	-	-
Shoulder dystocia	1.20 (0.82–1.74)	0.348	-	-

OR = odds ratio; CI = confidence interval.

**Table 6:** Absolute risk of neonatal adverse outcomes in pregnancies with gestational diabetes mellitus (GDM) compared to those with pre-existing DM and those without diabetes.

Neonatal Adverse Outcomes	Non-Diabetes (n = 49,122)	Pre-Existing DM (n = 509)	GDM (n = 2089)
Admission to NICU, n (%)	7104 (14.5)	267 (52.5) **	540 (25.8) **‡
Hypoxic ischaemic encephalopathy, n (%)	116 (0.2)	6 (1.2) **	6 (0.3) †
Hypoglycaemia, n (%)	493 (1.0)	103 (20.2) **	94 (4.5) **‡
Respiratory distress syndrome, n (%)	1618 (3.3)	88 (17.3) **	136 (6.5) **‡
Jaundice, n (%)	2541 (5.2)	134 (26.3) **	243 (11.6) **‡
Neonatal death, n (%)	34 (0.1)	2 (0.4) *	0 †

NICU = Neonatal intensive care unit; DM = Diabetes mellitus; Significance level p * and † *p* < 0.0167; ** and ‡ *p* < 0.001. Post hoc Bonferroni correction made for multiple comparisons. * Comparison of pre-existing DM and GDM with non-diabetic pregnancies; † Comparison between GDM and pre-existing DM.

**Table 7:** Univariable and multivariable logistic regression analysis demonstrating the association of gestational diabetes mellitus (GDM) with neonatal complications.

Neonatal Adverse Outcomes	Univariate Analysis		Multivariate Analysis	
	OR (95% CI)	*p* Value	OR (96%CI)	*p* Value
Admission to NICU	2.06 (1.86–2.28)	<0.001	1.71 (1.54–1.91)	<0.001
HIE	1.22 (0.54–2.77)	0.640	-	-
Hypoglycaemia	4.65 (3.71–5.82)	<0.001	3.67 (2.88–4.66)	<0.001
RDS	2.05 (1.71–2.45)	<0.001	1.59 (1.31–1.93)	<0.001
Jaundice	2.41 (2.10–2.78)	<0.001	2.05 (1.76–2.37)	<0.001
Neonatal death	-	-	-	-

NICU = Neonatal intensive care unit; HIE = Hypoxic ischaemic encephalopathy; RDS = Respiratory distress syndrome; OR = odds ratio; CI = confidence interval.

## Data Availability

Data are available from the authors upon request.

## Supplementary Materials

The following supporting information can be downloaded at: https://www.mdpi.com/article/10.3390/medicina59122096/s1, Supplementary Table S1. Univariate and multivariate logistic regression analysis demonstrating the association of pre-existing diabetes mellitus (DM) with antenatal pregnancy complications. Supplementary Table S2. Univariate and multivariate logistic regression analysis demonstrating the association of pre-existing diabetes mellitus (DM) with intrapartum pregnancy complications. Supplementary Table S3. Univariate and multivariate logistic regression analysis demonstrating the association of pre-existing diabetes mellitus (DM) with neonatal complications.
