# Supplementary material for: Gestational Diabetes Mellitus: Association with Maternal and Neonatal Complications
Source: Medicina (Kaunas). 2023 Nov 29;59(12):2096. doi: 10.3390/medicina59122096 (PMC10744613; doi:10.3390/medicina59122096)
Supplement: Supplementary file 1 [file medicina-59-02096-s001.zip › Supplementary Table S1.pdf]

Supplementary Table S1. Univariate and multivariate logistic regression analysis demonstrating the association of pre-existing diabetes mellitus (DM) with antenatal pregnancy complications.

Antenatal adverse outcomes	Univariate analysis		Multivariate analysis	
	**OR (95% CI)**	**P value**	**OR (95%CI)**	**P value**
Fetal defects - All	1.83 (1.21-2.77)	0.002	1.84 (1.21-2.80)	0.004
Central nervous system	3.35 (1.37-8.21)	0.008	3.42 (1.39-8.41)	0.007
Cardiovascular	4.73 (2.69-8.30)	<0.001	4.41 (2.50-7.78)	<0.001
Renal	0.94 (0.30-2.94)	0.915	-	-
Gastrointestinal	-	-	-	-
Musculoskeletal	0.85 (0.12-6.07)	0.868	-	-
Genetic	-	-	-	-
Fetal death				
Miscarriage	0.69 (0.26-1.84)	0.454	-	-
Stillbirth	5.72 (2.91-11.27)	<0.001	4.65 (2.31-9.35)	<0.001
Preterm delivery				
<32 weeks	4.39 (2.78-6.94)	<0.001	4.31 (2.65-7.01)	<0.001
<37 weeks	8.66 (7.20-10.41)	<0.001	7.98 (6.52-9.77)	<0.001
Fetal growth abnormalities				
SGA <10th percentile	0.60 (0.43-0.85)	0.004	0.57 (0.39-0.82)	0.003
LGA >90th percentile	5.40 (4.51-6.46)	<0.001	4.78 (3.87-5.90)	<0.001
Polyhydramnios		<0.001		<0.001
Mild	7.78 (5.95-10.18)	<0.001	3.99 (2.97-5.36)	<0.001
Moderate/severe	12.99 (6.21-27.18)	<0.001	6.75 (3.15-14.47)	<0.001
Obstetric complications				
Gestational hypertension	1.22 (0.65-2.30)	0.531	-	-
Preeclampsia	3.88 (2.82-5.33)	<0.001	2.97 (2.09-4.21)	<0.001

OR=odds ratio; CI=confidence interval; SGA=small for gestation; LGA=large for gestation
