# Supplementary material for: Gestational Diabetes Mellitus: Association with Maternal and Neonatal Complications
Source: Medicina (Kaunas). 2023 Nov 29;59(12):2096. doi: 10.3390/medicina59122096 (PMC10744613; doi:10.3390/medicina59122096)
Supplement: Supplementary file 1 [file medicina-59-02096-s001.zip › Supplementary Table S2.pdf]

Supplementary Table S2. Univariate and multivariate logistic regression analysis demonstrating the association of pre-existing diabetes mellitus (DM) with intrapartum pregnancy complications.

**Intrapartum adverse outcomes**	**Univariate analysis**		**Multivariate analysis**	
	**OR (95% CI)**	**P value**	**OR (95% CI)**	**P value**
Mode of delivery				
Unassisted vaginal	0.23 (0.19-0.28)	<0.001	0.30 (0.24-0.38)	<0.001
Operative vaginal	0.50 (0.33-0.75)	0.001	0.64 (0.42-0.98)	0.040
Elective caesarean section	4.07 (3.39-4.90)	<0.001	2.62 (2.12-3.23)	<0.001
Emergency caesarean section	2.37 (1.97-2.86)	<0.001	1.46 (1.17-1.82)	<0.001
Failure to progress	1.49 (1.08-2.05)	0.014	1.16 (0.83-1.63)	0.389
Fetal distress	2.56 (1.79-2.85)	<0.001	1.94 (1.51-2.50)	<0.001
Postpartum haemorrhage				
Moderate	1.88 (1.45-2.44)	<0.001	0.90 (0.68-1.18)	0.431
Severe	1.79 (1.02-3.11)	0.041	0.96 (0.54-1.69)	0.880
Obstetric anal sphincter injury	0.87 (0.41-1.84)	0.710	-	-
Shoulder dystocia				
All	0.67 (0.25-1.81)	0.433	-	-
Severe	2.14 (0.53-8.73)	0.287	-	-

OR=odds ratio; CI=confidence interval
