# Supplementary material for: Gestational Diabetes Mellitus: Association with Maternal and Neonatal Complications
Source: Medicina (Kaunas). 2023 Nov 29;59(12):2096. doi: 10.3390/medicina59122096 (PMC10744613; doi:10.3390/medicina59122096)
Supplement: Supplementary file 1 [file medicina-59-02096-s001.zip › Supplementary Table S3.pdf]

Supplementary Table S3. Univariate and multivariate logistic regression analysis demonstrating the association of pre-existing diabetes mellitus (DM) with neonatal complications

Neonatal adverse outcomes	Univariate analysis		Multivariate analysis	
	OR (95% CI)	P value	OR (96%CI)	P value
Admission to NICU	6.53 (5.47-7.78)	<0.001	4.39 (3.63-5.32)	<0.001
HIE	5.04 (2.21-11.51)	<0.001	5.09 (2.19-11.80)	<0.001
Hypoglycaemia	25.02 (19.81-31.61)	<0.001	12.29 (9.39-16.08)	<0.001
RDS	6.14 (4.85-7.76)	<0.001	2.39 (1.85-3.09)	<0.001
Jaundice	6.55 (5.36-8.01)	<0.001	4.10 (3.31-5.09)	<0.001
Neonatal death	5.70 (1.37-23.77)	0.017	3.19 (0.72-14.71)	0.127

OR=odds ratio; CI=confidence interval; NICU=Neonatal intensive care unit; HIE=Hypoxic ischaemic encephalopathy; RDS=Respiratory distress syndrome
